# SKA1 regulates actin cytoskeleton remodelling via activating Cdc42 and influences the migration of pancreatic ductal adenocarcinoma cells

**DOI:** 10.1111/cpr.12799

**Published:** 2020-03-30

**Authors:** Tong Li, Xu Liu, Bin Xu, Wei Wu, Yi Zang, Juanjuan Li, Lumin Wei, Yuting Qian, Hui Xu, Mingping Xie, Qi Wang, Lifu Wang

**Affiliations:** ^1^ Department of Gastroenterology Ruijin Hospital Affiliated to Shanghai Jiao Tong University School of Medicine Shanghai China

## Abstract

**Objectives:**

Spindle and kinetochore–associated protein 1(SKA1), originally identified as a protein essential for proper chromosome segregation, has been recently linked to multiple malignancies. This study aimed to explore the biological, clinical role and molecular mechanism of SKA1 in pancreatic carcinogenesis.

**Materials and Methods:**

SKA1 expression was detected in 145 pancreatic ductal adenocarcinoma (PDAC) specimens by immunohistochemistry. Biological behaviour assays were used to determine the role of SKA1 in PDAC progression in vitro and in vivo. Using isobaric tags for relative and absolute quantitation (iTRAQ), SKA1’s downstream proteins were examined. Moreover, cytochalasin B and ZCL278 were used to explore the changes of SKA1‐induced signalling and cell morphology, with further confirmation by immunoblotting and immunofluorescence assays.

**Results:**

Increased SKA1 expression was significantly correlated with tumour size and cellular differentiation degree in PDAC tissues. Furthermore, elevated levels of SKA1 reflected shorter overall survival (*P* = .019). As for biological behaviour, SKA1 acted as a tumour promotor in PDAC, overexpression of SKA1 facilitates cell proliferation, migration and invasion in vitro and in vivo. Mechanistically, we demonstrated that SKA1 enhanced pancreatic cancer aggressiveness by inhibiting G2/M arrest and regulating actin cytoskeleton organization via activating Cdc42.

**Conclusions:**

This study revealed novel roles for SKA1 as an important regulator of actin cytoskeleton organization and an oncogene in PDAC cells, which may provide insights into developing novel therapeutics.

## 1 INTRODUCTION

Pancreatic ductal adenocarcinoma (PDAC), as the predominant form of pancreatic cancer, accounts for 80%‐90% of all pancreatic cancer cases. According to the 2019 Cancer Statistics, PDAC has become the fourth leading cause of cancer‐related death (with <7% of patients surviving past 5 years), and even optimal treatments have a nominal influence on survival.^1^ PDAC is characterized by high malignancy and early metastatic potential, but the specific mechanisms involved are still elusive. Consequently, early detection and novel therapeutic strategies are urgently needed.

Epithelial‐mesenchymal transition (EMT) is the earliest event in tumour metastasis, and involves morphological changes of epithelial cells, accompanied by loss of cell polarity, disassembled tight junctions, looser cell‐cell adhesion and increased motility. All these events contribute to key molecular changes and are relevant to challenges of PDAC treatment.^2^ Understanding how a tumour cell experience EMT and establish itself at distant locations is indispensable, especially for cancers with early metastasis. One area of focus in EMT‐related cellular events has been the role of the dramatic cytoskeleton reorganization. The cytoskeleton is composed of three polymers, including microtubules, actin and intermediate filaments. The roles of actin and intermediate filaments in EMT are relatively well‐characterized: they facilitate cell structural plasticity, and alterations in the dynamics of the actin cytoskeleton have been implicated in cancer migration.^3^, ^4^, ^5^, ^6^ Microtubules, as the largest cytoskeletal components, govern intracellular trafficking, organelle positioning and molecular signalling, also affect the interactions with other cytoskeletal elements.^7^, ^8^ Hence, many anti‐microtubule agents (eg Taxanes) were derived, which increased survival in pancreatic cancer.^9^ However, their simultaneous deleterious activity towards normal cells is common because of low specificity, serious side effects are inevitable. Meanwhile, whether factors associated with microtubules as well as their associated proteins play a role in EMT of PDAC cells remains largely unknown. Therefore, we take this as the starting point to seek therapeutic targets with higher specificity and less toxicity.

Based on two independent microarray profiling datasets (TGF‐β stimulation‐induced EMT PANC‐1 cell line and control group) from the Gene Expression Omnibus and The Cancer Genome Atlas (TCGA) database. Spindle and kinetochore–associated protein 1 (SKA1) was focused on, because of its essential effects of promoting proper chromosome segregation and regulating the stability of both mitotic spindle and cytoskeletal microtubules in PDAC.^10^, ^11^, ^12^, ^13^ Recently, SKA1 was found to overexpressed in several malignancies and its oncogenic role has been demonstrated, including cell cycle distribution, chromosomal instability, cell proliferation and metastasis.^14^, ^15^, ^16^, ^17^ Additionally, SKA1 overexpression results in centrosome amplification in human prostate epithelial cells, which facilitates non‐tumorigenic epithelial cells switch to tumorigenic in nude mice,^18^ indicating that SKA1 may represent a master regulator of carcinogenesis. However, currently, the expression and molecular function of SKA1 in human pancreatic cancer remain undefined.

In this study, we determined the high expression and possible tumorigenic role of SKA1 in pancreatic cancer cells in vitro and in vivo, and the underlying mechanisms and signalling pathways were explored by iTRAQ assay. We firstly found that SKA1 overexpression induces Cdc42 activation, which in turn promotes actin cytoskeleton remodelling, providing a potential therapeutic strategy to overcome PDAC.

## 2 MATERIALS AND METHODS

### 2.1 Patients and the clinical cohort

A total of 145 formalin‐fixed and paraffin‐embedded sections of human PDAC specimens were retrospectively collected from patients who underwent pancreatectomy without preoperative radiation or chemotherapy in Ruijin Hospital (Shanghai, China) before 2019. Histological diagnosis was performed by two experienced pathologists independently. This study was approved by the ethics committee of Ruijin Hospital.

### 2.2 Cell lines, reagents and inhibitors

Human pancreatic cancer cell lines (CFPAC‐1, Capan‐1, Capan‐2, HPAF‐II, BxPC‐3, ASPC‐1 and PANC‐1) were obtained from American Tissue Type Culture Collection (ATCC). hTERT‐HPNE, SW1990 and MIA PaCa‐2 were obtained from the Cell Bank of the Chinese Academy of Science. All cells were grown in Dulbecco's modified Eagle's medium (DMEM, Hyclone) supplemented with 10% foetal bovine serum (Gibco), 4 mmol/L l‐glutamine, 4500 mg/L glucose, sodium pyruvate, 100 μg/mL streptomycin and 100 U/mL penicillin, at 37°C in an atmosphere with 5% CO_2_. ZCL278 (Cdc42 inhibitor) was purchased from Absin (abs812880); cytochalasin B (inhibitor of F‐actin polymerization) was purchased from Meilunbio (MB5434).

### 2.3 Lentivirus‐mediated stable transfection

SKA1 interference and overexpression lentiviruses were constructed with GV208 by GeneChem Company. PDAC cells were seeded into 6‐well plates, and lentiviral transfection with experimental constructs or respective controls (1 × 10^9^ TU/mL) was carried out at 60% confluency, according to the manufacturer's protocol. After 48‐72 hours, infection efficiency was detected and SKA1 expression levels were verified. To establish stably transfected cells (sh‐SKA1, sh‐ctr, SKA1 and vector), cells were further cultured in 10 μg/mL puromycin containing medium.

### 2.4 Cell proliferation, migration and invasion assays

For the cell proliferation MTT assay, detailed procedures are provided as we have previously described.^19^ Transwell migration assay was performed using transwell chambers (24‐well, 8 μm pore size; Corning), and cell invasion assay was conducted with BD Falcon Cell culture inserts coated with BD matrigel matrix (BD Bioscience). About 2 × 10^5^ cells were seeded in the upper chambers with DMEM, and DMEM supplemented with 10% FBS was added to the lower chambers. The plates were incubated at 37°C for 24 hours. Cells that did not migrate or invade through the pores were removed with cotton swabs. Cells on the lower side of the filter were fixed with 10% formalin and stained with crystal violet, finally counted to evaluate the migratory and invasive abilities.

### 2.5 Wound‐healing assay

Wound‐healing assays were performed in 6‐well plates with confluent cells, with scratches generated using 200 μL pipette tips. The wells were then washed three times with the culture medium and cultured for an additional 24 or 48 hours, followed by the assessment of relative wound closure areas.

### 2.6 Flow cytometry

3 × 10^5^ cells with stable lentiviral transfection were transferred into 0.5 mL cold PBS followed by fixation with 70% ethanol at 4°C for 24 hours. The fixed cells were pelleted at 1500 rpm for 5 minutes and washed twice with PBS. Next, the cells were incubated with RNase supplemented propidium iodide (PI) (Servicebio) in PBS at 37°C for 30 minutes. For cell cycle analysis, cell suspensions were transferred into sterile polystyrene tubes. The percentages of cells in various cell cycle phases (eg G1, S and G2/M) were analysed on a flow cytometer (CytoFLEX, Beckman Coulter).

### 2.7 Immunoblotting and quantitative real‐time PCR (qRT‐PCR)

Immunoblotting procedures were described in our previous study.^20^ Cytoskeletal fractions were obtained using a ProteoExtract Subcellular Proteome Extraction Kit (Merck Millipore) according to the manufacturer's instruction. Primary antibodies are shown in Table S1. Immunoreactivity was detected by the Enhanced Chemiluminescence kit (GE Healthcare), and visualization was performed with the G BoxChemic XL system (Syngene). GAPDH was used as an internal reference control for the relative protein expression levels using the Quantity One software (Bio‐Rad). For qRT‐PCR, total RNA was isolated with TRIzol reagent (Invitrogen), and cDNA was synthesized with the First Strand cDNA Synthesis kit (Yeasen), following the manufacturer's instructions. For quantitative reverse transcriptase‐PCR, SYBR Green Supermix kit (Yeasen) was used on a StepOnePlus™ Real‐Time PCR System (Applied Biosystems). Primer sequences for qRT‐PCR were as follows: SKA1 forward: ACCCAGAGCTGTGTTAAGGGA, SKA1 reverse: TTGGGAGGCTTCTTT ACGGGT; GAPDH forward: GGTGAAGGTCGGAGTCAACG, GAPDH reverse: TGGGTGGAATCATATTGGAACA. The relative gene expression levels were determined by the comparative CT method (2^−ΔΔCT^). The results were expressed as relative integrated intensity.

### 2.8 Cdc42‐GTP pull‐down assay

Cdc42 activation was examined using the Cdc42 Activation Assay Kit (ab211163, Abcam) following the manufacturer's instructions. Cells were harvested with cell lysis buffer, 293 cell lysate loaded with GDP or GTPγS and incubated with PAK1 PBD Agarose beads were as negative or positive controls, respectively. One mg of protein lysate in a 1 mL total volume at 4°C was immediately precipitated with 40 μL of PA1K‐PBD beads for 60 minutes with rotation. After washing, beads were resuspended and processed for immunoblotting.

### 2.9 Histology, immunocytochemistry and immunofluorescence

Immunohistochemical experiment was operated using standard techniques according to our previous study.^21^ To evaluate SKA1 expression levels, a semi‐quantitative immunoreactivity score analysis method scoring both the percentage of positive cells and staining intensity was used. The percentage of positive cells was scored from 0 to 3 (0, 0%; 1, 1%‐49%; 2, 50%‐70%; and 3, >70%); staining intensity was scored from 0 to 3 (0, negative; 1, weak staining; 2, intermediate staining; and 3, strong staining). The two sub‐scores were added to determine the final score. We defined the samples having a final score ≤ 3 as low expression and samples, with a score of >3 as high expression. Each slide was evaluated double‐blindedly by two experienced pathologists independently. For Immunofluorescence staining, procedures were described in our previous study,^22^ and primary antibodies are shown in Table S1. All slides were examined by confocal microscopy (Zeiss710), and photometric values were analysed by ImageJ.

### 2.10 In vivo subcutaneous xenograft and metastasis assay

All mouse care and handling procedures were conducted in accordance with the recommendations of the Institutional Animal Care and Use Committee of Shanghai Experimental Animals Centre, Chinese Academy of Sciences. Briefly, PANC‐1 cells as well as Capan‐1 cells stably transfected with sh‐SKA1, sh‐ctr, SKA1 or empty vector were washed in 0.15 mL serum‐free DMEM. In xenografts’ assay, 3 × 10^6^ cells were subcutaneously transplanted into the right or left flank of 4‐ to 5‐week‐old female BALB/c nude mice (n = 5/group), and tumour volume was monitored until the mice were sacrificed 30 days later. For metastasis analysis, 1 × 10^6^ cells were intravenously injected into the tail vein to study lung metastasis (n = 10/group); 12 weeks later, the mice were euthanized, and lung samples stained with haematoxylin and eosin were evaluated under a microscope.

### 2.11 iTRAQ and bioinformatic analysis

We performed Isobaric Tags for Relative and Absolute Quantitation (iTRAQ) with stably transfected sh‐SKA1 and sh‐ctr in PANC‐1 cells. After protein extraction and trypsin digestion, the peptides were desalted with a Strata X C18 SPE column (Phenomenex) and vacuum dried. Peptides were reconstituted in 0.5 mol/L TEAB and processed according to the manufacturer's protocol for TMT/iTRAQ kit. Briefly, one unit of TMT/iTRAQ reagent was thawed and reconstituted in acetonitrile. The peptide mixtures were then incubated for 2 hours at room temperature and pooled, desalted and dried by vacuum centrifugation. Subsequently, bioinformatic analyses (GO annotation, KEGG pathway annotation, protein‐protein interaction network, etc) were performed.

### 2.12 Statistical analysis

Statistical analyses were performed with the SPSS 20.0 software (SPSS Inc). Excel and GraphPad Prism version 6 (GraphPad Software) were employed to process the initial data and plot graphs. Student's *t* test and one‐way analysis of variance were performed to compare groups. The correlation between SKA1 amounts and Cdc42 expression in PDAC tissue samples was evaluated by Pearson's correlation analysis. A significance level of *P* < .05 was assumed for all statistical evaluations (*P < .05 and **P < .01). Data are presented as the mean ± SEM from three independent experiments unless otherwise noted.

## 3 RESULTS

### 3.1 Increased SKA1 is significantly correlated with poor survival outcomes in PDAC

**Figure 1:**
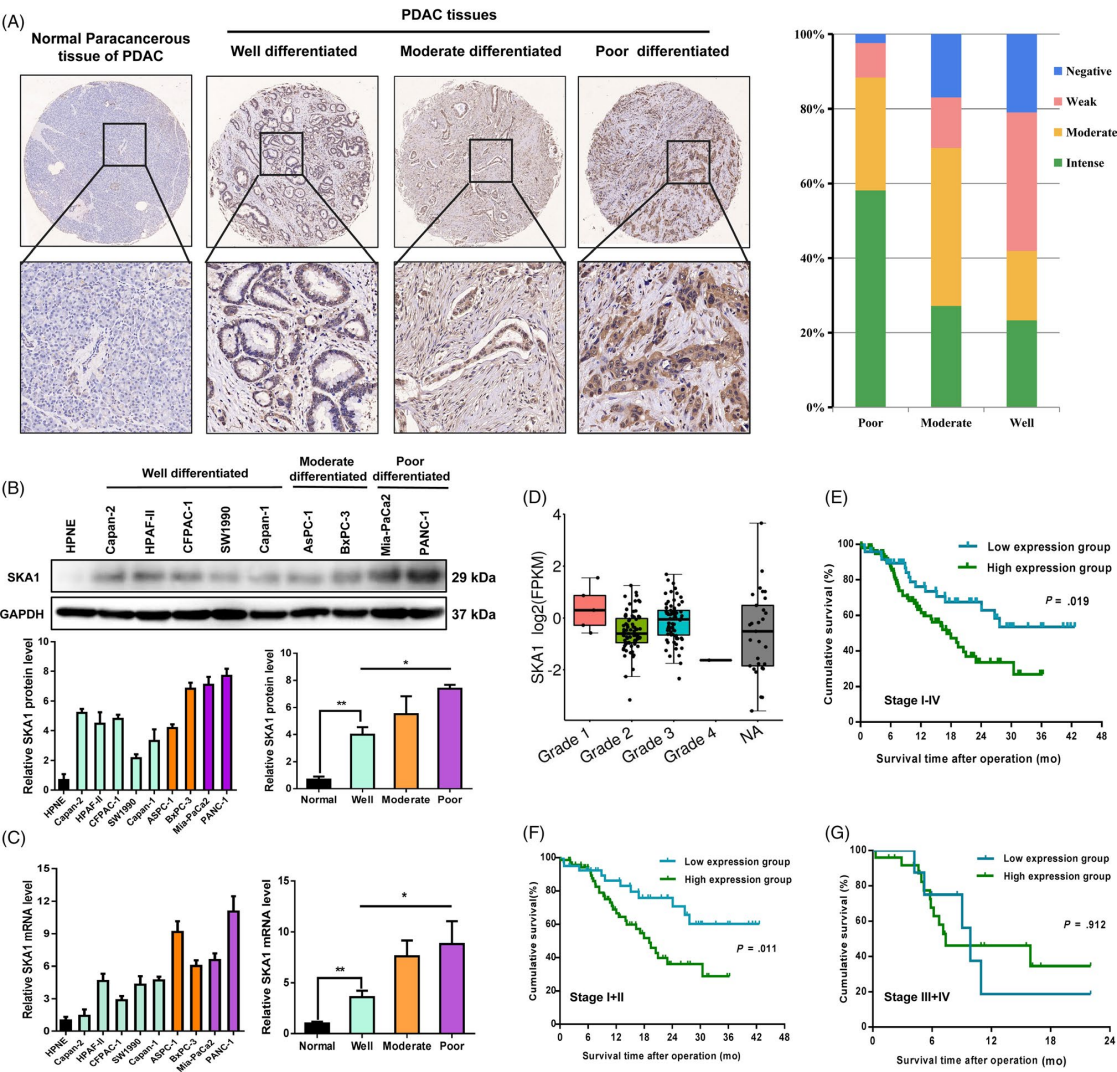
SKA1 is overexpressed in PDAC and associated with differentiation level and poor prognosis. A, Representative immunohistochemistry staining of different degrees of differentiation in 145 PDAC patients’ tissues for SKA1 is shown (left), and statistical histogram shows that the expression level SKA1 was increased along with poorer differentiated PDAC (right). B and C, Expression of SKA1 in one normal pancreatic duct epithelial cell line (hTERT‐HPNE), five well‐differentiated cell lines (Capan‐2, HPAF‐II, CFPAC‐1, SW1990 and Capan‐1), two moderate differentiated cell lines (AsPC‐1 and BxPC‐3) and two poor differentiated cell lines (MIA PaCa‐2 and PANC‐1) was measured by Immunoblotting and qRT‐PCR, respectively. GAPDH was used as the loading control. D, No significance of SKA1 expression level was observed among different stages in TCGA database. E‐G, Kaplan‐Meier survival curves of overall survival according to SKA1 expression in 145 PDAC patients of the testing cohort were performed using the log‐rank test. High SKA1 expression predicts poor overall survival in PDAC patients (*P* = .019), especially for patients with relative early TNM stage (*P* = .011), but not for late TNM stage (*P* = .912)

SKA1 (identified as EMT‐related dysregulated protein in PDAC cells, see Figure S1) is expressed in various types of tumours; however, whether SKA1 expression is involved in pancreatic cancer development remains unknown. According to our immunohistochemical results, SKA1 staining was positive in 97 (66.9%) PDAC cancer samples, while no or weak staining was observed in 48 cases (33.1%). Risk factors for PDAC did not significantly differ between the two groups, nor did gender and age. Interestingly, patients with high SKA1 expression tended to present greater tumour diameters compared with the low SKA1 expression group (*P* = .019) (Table 1). Furthermore, we found that SKA1 expression was significantly correlated with PDAC cellular differentiation degree (Figure 1A).

**Table 1:** Baseline characteristics of PDAC patients stratified by SKA1 protein expression

Clinical parameters	Overall (n = 145)	SKA1 protein expression		*P* value
		Low (n = 48)	High (n = 97)	
Gender				
Male	82	27	55	.959**
Female	63	21	42	
Age (y)				
Mean ± SD	66.30 ± 9.431	65.59 ± 10.07	66.67 ± 9.12	.602*
>65(%)	55.2	52.1	56.7	.413**
Grade				
G1	102	33	69	.767**
G2	43	15	28	
T classification				
T1 + T2	66	20	46	.512**
T3 + T4	79	28	51	
N classification				
Absent	94	31	63	.207**
Present	17	3	14	
Metastasis				
Yes	7	2	5	1***
No	138	46	92	
Tumour location of pancreas				
Head and neck	99	29	70	.153**
Body and tail	46	19	27	
Nerve infiltration				
Yes	98	28	70	.094**
No	47	20	27	
Vessel infiltration				
Yes	27	6	21	.183**
No	118	42	76	
Tumour diameter (cm)				
≤3	69	31	38	.019**
>3	76	20	56	

* Independent sample test.

** Pearson chi‐squared test.

*** Continuity correction.

Next, SKA1 expression levels were assessed in 1 normal (hTERT‐HPNE), 5 well (SW1990, Capan‐1, Capan‐2, CFPAC‐1 and HPAF‐II), 2 moderately (BxPC‐3 and ASPC‐1) and 2 poorly (MIA PaCa‐2 and PANC‐1) differentiated PDAC cell lines by immunoblotting. As shown in Figure 1B, varied expression levels were observed in these cell lines, with maximum amounts in PANC‐1 and BxPC‐3 cells, followed by the remaining poorly and moderately differentiated cell lines. In contrast, the 5 well‐differentiated PDAC cell lines exhibited relatively low SKA1 expression levels. mRNA expression patterns were approximately consistent with immunoblotting profiles (Figure 1C). These findings were consistent with the above histological differentiation results that elevated SKA1 expression was correlated with poor cancer cell differentiation. Nevertheless, no significance was observed among different stages in the TCGA database (Figure 1D).

In addition, 145 PDAC patients with higher SKA1 levels had shorter overall survival time compared with those expressing lower (*P* = .019), especially for cases with relative early TNM stage (stage I,II) (*P* = .011), but not for late TNM stage ones (stage III,IV)(*P* = .912) (Figure 1E‐G). Finally, univariate and multivariate Cox regression analyses were performed to identify SKA1 as an independent prognostic marker for PDAC‐specific overall survival (Table 2).

**Table 2:** Predictive factors for overall survival in univariate and multivariate logistic regression analyses

	Univariate			Multivariate		
	HR	95% CI	*P* value	HR	95% CI	*P* value
SKA1	1.987	1.110‐3.560	.021	2.07	1.148‐3.731	.016
Grade	2.028	1.190‐3.456	.009	1.925	1.131‐3.278	.018
T classification	1.497	1.097‐2.043	.011	1.141	0.711‐1.833	.584
Metastasis	3.696	1.229‐10.515	.014	2.926	1.024‐8.363	.045
Vessel infiltration	2.202	1.170‐4.144	.014	1.455	0.548‐3.863	.452
Tumour diameter	1.959	1.150‐3.336	.013	2.035	1.154‐3.587	.014

### 3.2 SKA1 enhances PDAC proliferation in vitro and in vivo by inhibiting G2/M arrest

**Figure 2:**
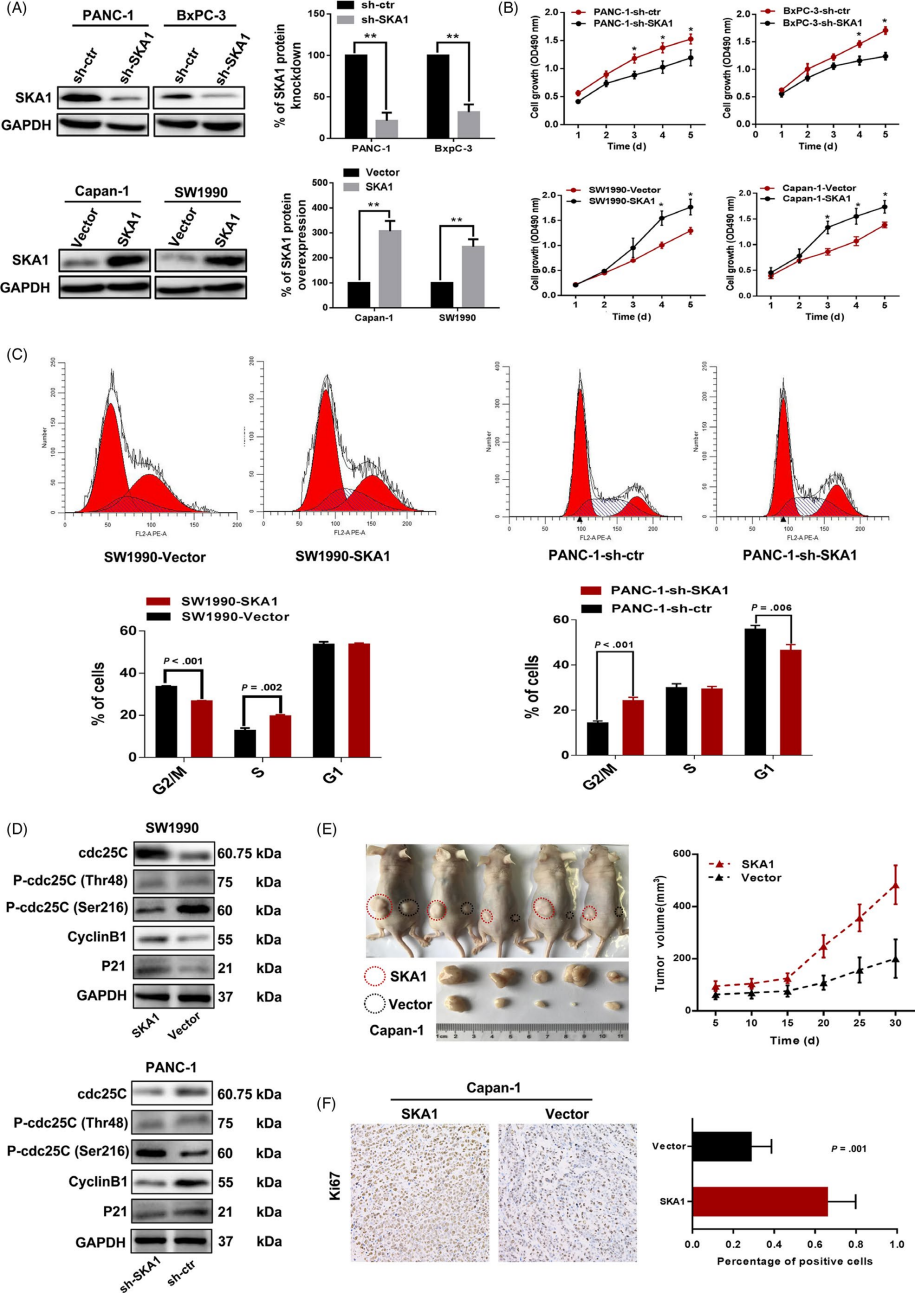
SKA1 promotes PDAC proliferation in vitro and in vivo. A, Immunoblotting was performed in PANC‐1 and BxPC‐3 cells transfected with control shRNA (sh‐ctr) and SKA1 knock‐down shRNAs (sh‐SKA1), in Capan‐1 and SW1990 cells transfected with empty vector (vector) and lentivirus‐mediated flag‐tagged overexpression SKA1(SKA1). B, MTT assay showed the SKA1 facilitates PDAC cell growth ability, the significances were identified based on the comparison of counterpart. *P < .05. C and D, Cell cycle analysis by flow cytometry presented a significantly increased percentage of sh‐SKA1 cells in the G2/M phase, and related proteins were detected by Immunoblotting. E, The subcutaneous tumorigenic ability of tumour cells was measured (n = 5 per group). Expression of SKA1 promoted tumour growth and increased tumour weight in nude mice (*P* = .03). F, Percentage of positive Ki67 staining cells in tumour tissues was counted by immunohistochemical analysis. Data are presented as the mean ± SEM from three independent cell function experiments

Since higher SKA1 expression levels were associated with worse prognosis, and an increasing expression trend was found in larger size PDAC tissue samples, we hypothesized that SKA1 might play an inductive role in PDAC growth. We selected PANC‐1 and BxPC‐3 cells (highest SKA1 levels as shown above) to perform SKA1 knock‐down, and Capan‐1 and SW1990 cells (lowest amounts as shown above) for overexpression, respectively (Figure 2A), to examine its biological functional significance in PDAC cell growth. We first investigated the impact of SKA1 knock‐down on cell proliferation by the MTT assay, and significant growth inhibition was observed in BxPC‐3 and PANC‐1 cells compared with vehicle‐treated cells (*P* < .05); overexpression of SKA1 in Capan‐1 and SW1990 cells had opposite effects (Figure 2B). In addition, SKA1 promotes PDAC cells proliferation was also evidenced by colony formation and cell apoptosis assays (Figure S2).

Next, we examined cell cycle distribution by flow cytometry; significantly, increased amounts of PANC‐1‐sh‐SKA1 cells were found in the G2/M phase (*P* < .001), indicating that SKA1 depletion was potentially associated with G2/M arrest (Figure 2C). To elucidate its molecular basis, G2/M arrest‐associated proteins were investigated. Results showed that knock‐down of SKA1 lead to G2/M arrest by phosphorylating cdc25C (Ser216) and regulating the p21, cyclinB1 in PANC‐1 cells, and vice versa in SW1990 cells (Figure 2D). These findings suggested that SKA1 increases proliferation by promoting G2/M cell cycle progression.

Finally, to evaluate the in vivo effect of SKA1, we performed subcutaneous xenograft assays in nude mice, and SKA1 overexpression significantly increased tumour growth, along with a marginally increased expression of Ki67 (Figure 2E,F). Likewise, similar results were obtained in PANC‐1 cells **(**Figure S2).

### 3.3 Loss of SKA1 suppresses migration and invasion and confers resistance to EMT

It is universally acknowledged that EMT is one of the most important factors associated with three major steps (invasion, dissemination and metastasis) in pancreatic cancer.^23^ Due to the fact that poorly differentiated cancer cells are more prone to early metastasis, and poorly differentiated pancreatic cancer tissues/cells showed higher SKA1 expression levels than well‐differentiated counterparts (see above), whether SKA1 facilitates migration and invasion in PDAC cells is an interesting question.

**Figure 3:**
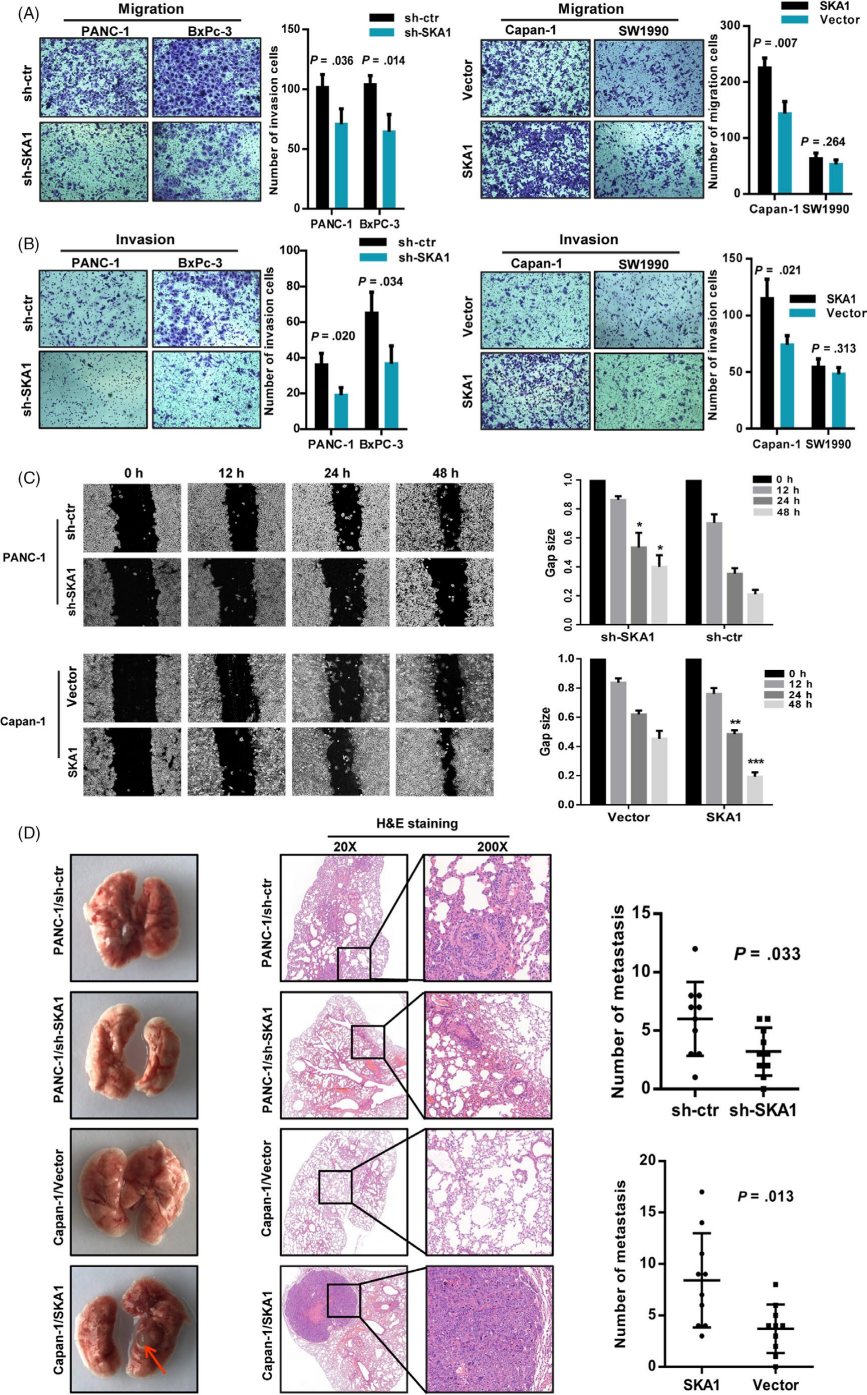
SKA1 accelerates tumour invasion and metastasis via EMT. A and B, Transwell migration and Matrigel invasion assay. sh‐SKA1 infectants exhibited significantly reduced migration and invasion capacity than the sh‐ctr infectants, except for SW1990 cell line. C, Wound‐healing assay measured the effect of SKA1 on PDAC cell motility. Left: Representative images of scratched and recovering of wounded areas taken at different time points. Right: Semi‐quantitative analysis of gap size by ImageJ. The significances were calculated based on the comparison of the control group. *P < .05; **P < .01; ***P < .001. D, Representative haematoxylin‐eosin staining and summarized data on number of metastasis of lung in nude mice at 12 wk after tail vein injection of PANC‐1 or Capan‐1 cells with SKA1 knock‐down or overexpression (n = 10 per group), red arrow, metastatic nodule (*P* = .033, *P* = .013, respectively)

We evaluated the effect of SKA1 on the malignant phenotype of PDAC cells in vitro. Results showed that knock‐down of SKA1 markedly inhibited cell migration and invasion in PANC‐1 and BxPc‐3 cells, and its overexpression notably promoted migration and invasion in Capan‐1 cells, except for SW1990 cells (Figure 3A,B). These results were further validated by wound‐healing assays. Indeed, consistent with the transwell experiments results, PANC‐1‐sh‐SKA1 cells filled approximately 55% of the scratched wounds in a time period of 24 hours, whereas PANC‐1‐sh‐ctr cells showed more than 80% motility under these conditions, and vice versa in Capan‐1 cells (Figure 3C).

**Figure 4:**
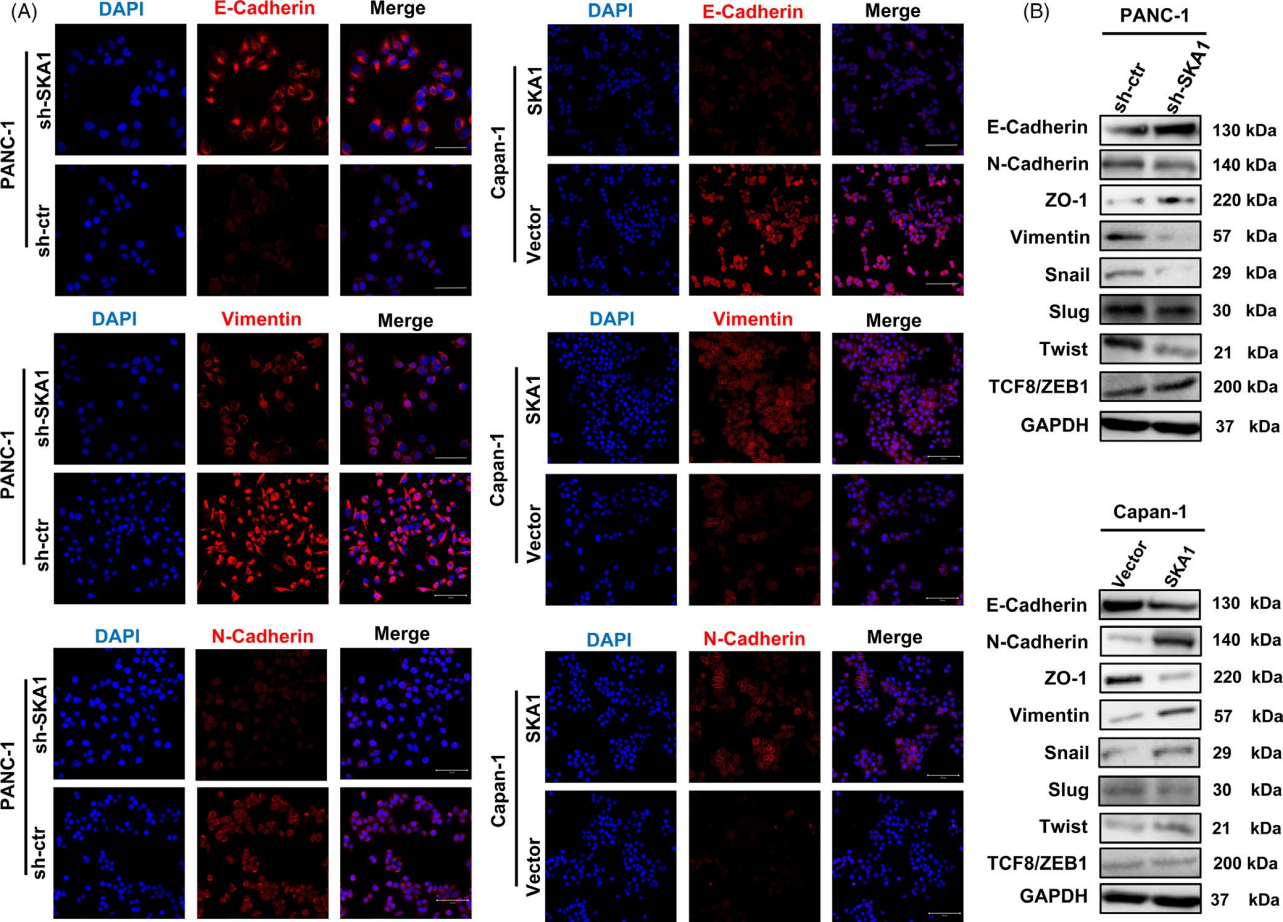
SKA1 facilitates EMT in PDAC cells. A, A significant reduction of vimentin and N‐cadherin but increase E‐cadherin was detected in PANC‐1‐shSKA1 cells compared to PANC‐1‐sh‐ctr cells by immunofluorescence staining, and vice versa in Capan‐1 cells. B, The protein expression levels of EMT markers and transcription factors (E‐cadherin, N‐cadherin, ZO‐1, Vimentin, Snail, Slug, Twist, TCF8/ZEB1) in modified PANC‐1 and Capan‐1 cells were assayed by Western blotting. GAPDH was used as an internal control

For in vivo PDAC metastatic models, tail vein metastasis assay demonstrated that SKA1 significantly enhanced the number of metastasis of lung in nude mice at 12 weeks (Figure 3D). And there were fewer metastatic foci for SKA1‐knock‐down group. Immunofluorescence also revealed that SKA1 overexpression led to increased vimentin and N‐ cadherin signals but decreased E‐cadherin immune reactivity (Figure 4A). To understand the mechanism by which SKA1 reduces E‐cadherin expression, we checked the expression of EMT regulatory transcription factors. As shown in Figure 4B, SKA1 overexpression significantly increased the level of Snail and Twist, whereas these protein levels were decreased in SKA1‐knock‐down cells, suggesting that SKA1 may facilitate EMT via Snail and Twist.

Taken together, these data implied that depletion of SKA1 likely suppressed the migration‐invasion cascade of PDAC.

### 3.4 Comparative proteome‐wide analysis of SKA1‐induced biological processes

**Figure 5:**
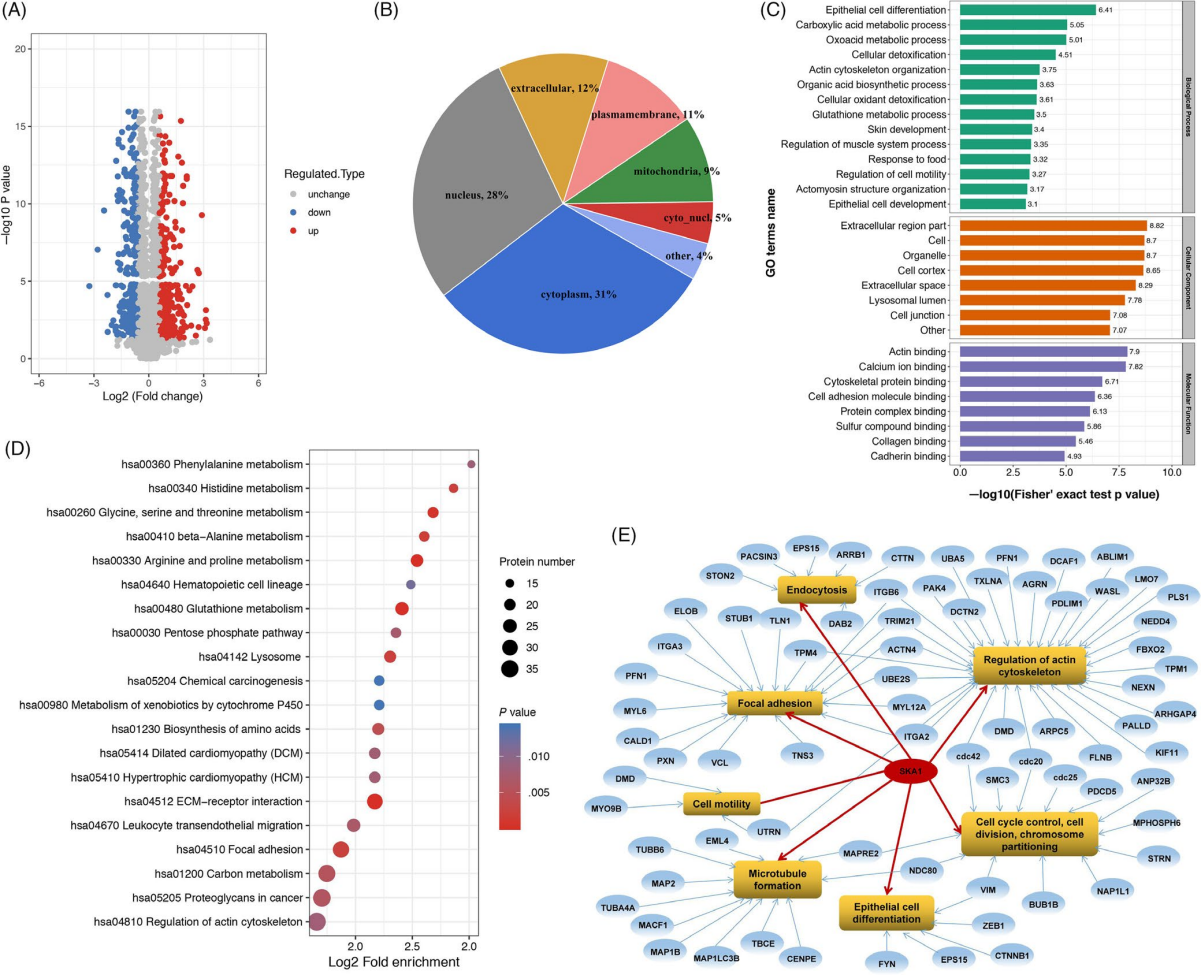
Proteomic analysis of SKA1‐induced biological process. A, Volcano plot showing the quantitative protein expression from sh‐SKA1 and sh‐ctr in PANC‐1 cells. Proteins differentially expressed with fold change over 1.5 were marked in blue and red. B, Subcellular distribution of the differentially expressed proteins. C, The top enriched biological processes, cellular components and molecular functions based on the gene ontology (GO) analysis with the sequence data of differentially expressed proteins. D, Bubble chart that illustrate the top 20 signalling pathways enriched based on the KEGG pathway analysis. E, Network of gene ontology and related differential proteins

Since SKA1 exerted obvious effects for PDAC, we conjectured that more detailed and concrete pathways as well as potential effectors must underlie this phenomenon. To further unveil this mechanism, PANC‐1 cells stably transfected sh‐SKA1 and sh‐ctr were generated as in vitro models to perform iTRAQ analysis. A total of 5351 proteins were obtained, with 868 identified as differentially expressed between groups with a cut‐off fold change of 1.5 and *P* < .05. Of these, 448 proteins were upregulated and 420 were downregulated (Figure 5A). Subcellular distribution of the differentially expressed proteins was annotated as Figure 5B.

Next, in order to obtain an overview of the functions of differentially expressed proteins, GO functional classification was conducted based on biological processes, cellular component and molecular function. Within the category of molecular function, the most significant group of GO annotated proteins was involved in actin binding activities, mainly including but not limited to calcium ion binding, cytoskeletal protein binding and cell adhesion molecule binding. According to biological processes, functions in epithelial cell differentiation were significantly enriched (Figure 5C). Moreover, enrichment analysis (*P* < .01) using the KEGG was performed to investigate the possible roles that were preferred targets of downstream pathways by SKA1 regulation. As shown in Figure 5D, the largest group of differential proteins was involved in regulation of actin cytoskeleton, followed by proteoglycans in cancer, which as a major component of the ECM can interact with numerous regulators of cell behaviour through signalling to the actin cytoskeleton and cell adhesion.^24^, ^25^ Additionally, major GO terms including the related annotated proteins were also presented as a PPI network (Figure 5E), which highlighted the possible intracellular signalling pathways, consequently generating the concomitant onset of PDAC cell activities (eg cell proliferation, migration and invasion) and providing a promising starting point for further exploring the mechanisms of SKA1‐induced biological processes.

### 3.5 Insights into SKA1‐modulation of cell morphology and actin cytoskeleton in PDAC

**Figure 6:**
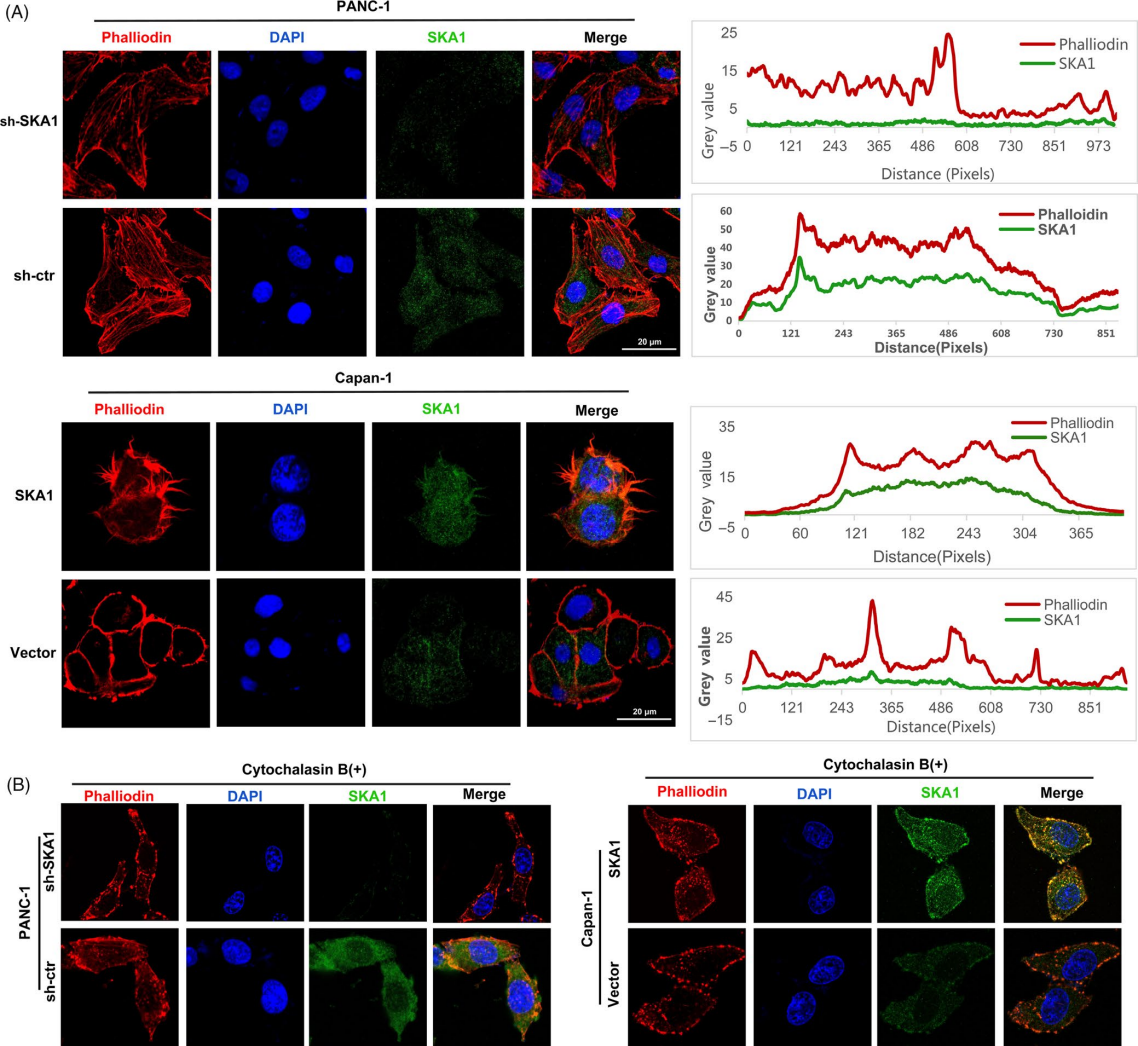
SKA1 modulates the formation of cytoskeletal actin filament and shared partial co‐localization with F‐actin in PDAC cells. A, Combined rhodamine‐phalloidin staining for F‐actin (red) and SKA1 (green) immunofluorescence, nuclei were counterstained with DAPI (blue). Upper two panels: stable knock‐down of SKA1 reduced actin protrusions and stress fibres in PANC‐1 cells. Lower two panels: stable overexpression of SKA1 altered the cellular morphology and lead to longer invadopodia formation compared with vector group in Capan‐1 cells. Additionally, SKA1 and phalloidin‐stained F‐actin shared partial co‐localization to the cytoplasm. Co‐localization correlation of intensity distributions curves between two channels was drawn by ImageJ and measured by Pearson's correlation coefficient analysis (right). B, With cytochalasin B treatment, F‐actin filaments stained with phalloidin were not seen in both PANC‐1 and Capan‐1 cells’ infectants

Based on the above analysis, actin cytoskeleton regulation weighs highly in SKA1‐induced biological processes. The actin cytoskeleton is formed by polymerization of actin monomers (G‐actin) to generate microfilaments (F‐actin) and represents a highly dynamic structure that involves continuous assembly and turnover of essential cellular processes, including endocytosis, intracellular transport, cell morphogenesis and cell motility. The reorganization of the actin cytoskeleton and the formation of migratory membrane protrusions are the key mechanical drives for morphological changes and gained invasive properties of metastatic cancer cells.^26^ Hence, we performed immunofluorescent staining of SKA1 and phalloidin on F‐actin organization. Coincidentally, we noted that SKA1 shared partial co‐localization with F‐actin in PDAC cells; besides, a striking alteration in the morphology of cultured PANC‐1 cells stably knocked down for SKA1, which showed less stress fibres or F‐actin filaments and less filopodia and lamellipodia than control cells, consistent with a shift from a classic mesenchymal phenotype to a more epithelial one. Conversely, results also showed that overexpression of SKA1 could alter the cellular morphology and lead to longer invadopodium formation (Figure 6A).

To examine whether this phenomenon can be altered or disrupted by cytoskeletal‐directed agents, we use cytochalasin B (a inhibitor of F‐actin polymerization) for treatment. As expected, cells are profoundly sensitive to cytochalasin B with the microfilaments depolymerize to a great extent in SKA1‐overexpressing, SKA1‐knock‐down and control groups in both PANC‐1 and Capan‐1 cells (Figure 6B), which suggested that cytochalasin B could abrogate the formation of F‐actin in PDAC cells potently but unspecifically, and damage may be attainable with this treatment protocol.

### 3.6 SKA1 remodels the actin cytoskeleton via activating Cdc42

**Figure 7:**
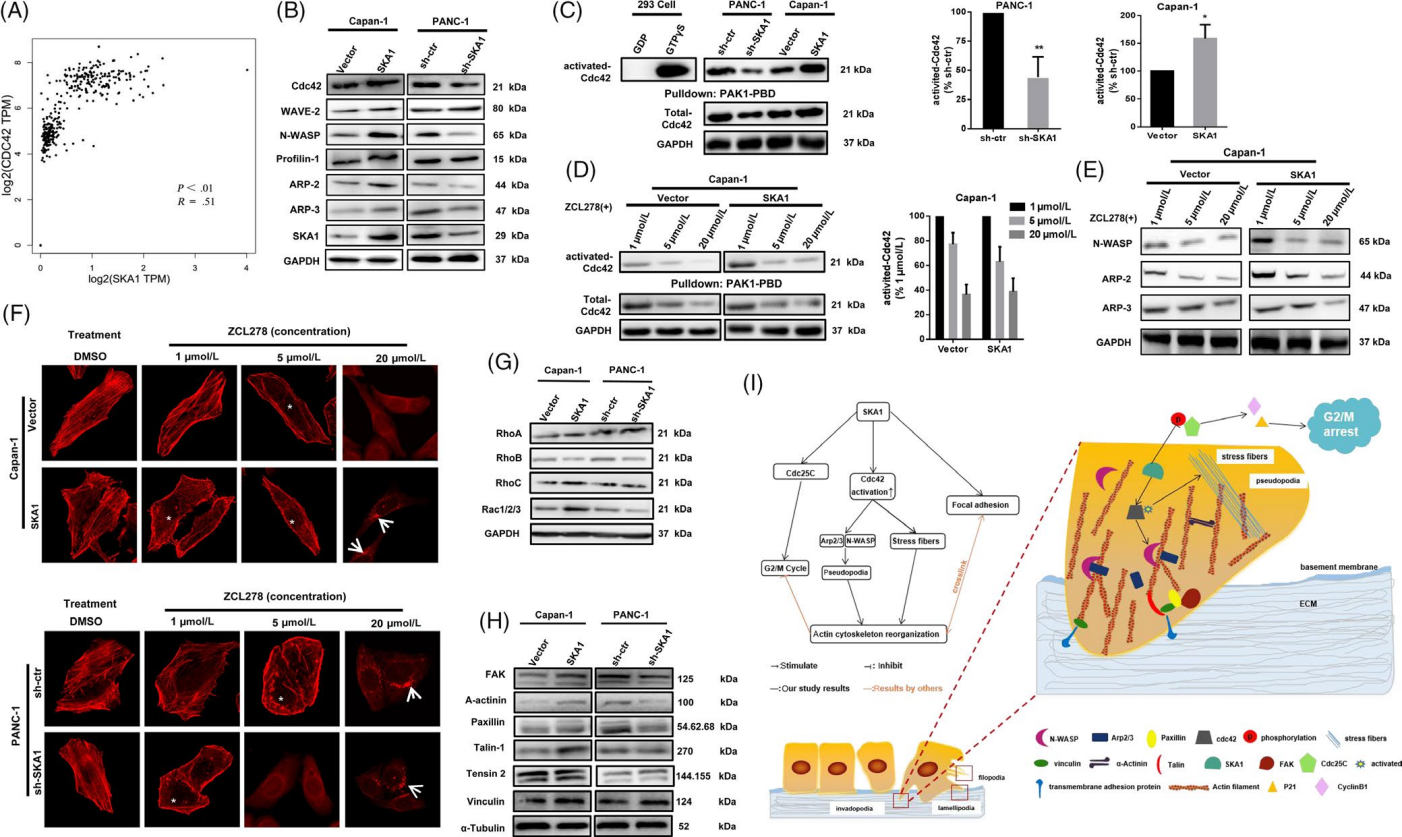
SKA1 remodels actin cytoskeleton via activating Cdc42. A, Pearson's correlation test was used to analyse the correlation between the expression level of SKA1 and Cdc42 according to TCGA database (*R* = .51, *P* < .01). B, Immunoblotting performed in PANC‐1 and Capan‐1 cells’ infectants to verify the proteins that regulate actin cytoskeleton. C, Cdc42‐GTP pull‐down and Western blot analyses of the activation state of Cdc42 expression and total expression of Cdc42 in the indicated cells. D and E, The application of ZCL278 resulted in a dose‐dependent decrease in Cdc42 activity, followed by inhibition of endogenous Arp2/3, N‐WASP. F, Cells were fixed and stained with rhodamine‐phalloidin to label filamentous actin following ZCL278 treatments. Results showed that ZCL278 inhibits microspike formation and stress fibres in PANC‐1 and Capan‐1 cells. White asterisks indicate the subcellular locations that normally show stress fibre distribution. White arrows point to the seemingly disruption Golgi organization. G, RhoA, RhoB, RhoC and Rac1/2/3 protein expression were detected, and only a total of Rac1/2/3 increase accompanied by SKA1 overexpression was observed. H, FAK, Talin and ɑ‐actinin levels increased with SKA1 expression in Capan‐1 cells, FAK, Paxillin and ɑ‐actinin levels decreased with SKA1 knockout in PANC‐1 cells. I, Proposed mechanistic scheme of SKA1 in promoting tumour progression in PDAC

What are the specific molecular mechanisms leading to the occurrence of the above phenomenon? By protein functional prediction and differential protein expression folds analysis, we hypothesized that the biological processes induced by SKA1 in PDAC cells could be mediated through regulation of Cdc42 (with a cut‐off fold change of 5.4 and *P* = 7.8629E‐09), which could facilitate filopodia formation, regulate cell migration and regulate bipolar attachment of spindle microtubules to kinetochores in metaphase.^27^, ^28^, ^29^ We first did the correlation analysis between SKA1 and Cdc42, as shown in Figure 7A, and Cdc42 expression was significantly positively correlated with SKA1 amounts in PDAC tissues in TCGA database, with a Pearson's correlation coefficient of *R* = .51 (*P* < .01). Besides, PAK1‐PBD pull‐down and immunoblotting were performed to verify the above speculation. Results showed SKA1 was associated with Cdc42 activation, and increased levels of N‐WASP and Arp2/3 subsequently, but not WAVE‐2 and profilin‐1. Implying that the actin cytoskeleton remodelling by SKA1 overexpression in PDAC cells was likely occurred via its association with Cdc42 activation (Figure 7B,C).

Moreover, we used ZCL278, a compound that directly binds to Cdc42 and displays most inhibitory effects in a morphological assay of Cdc42 function, to assess its activity at the biochemical level by incubation at concentrations of 1, 5 and 20 μmol/L for 10 minutes, respectively. As depicted in Figure 7D,E, application of ZCL278 resulted in a dose‐dependent decrease in Cdc42 activity, total Cdc42 level as well as N‐WASP and Arp2/3 levels. Meanwhile, ZCL278‐treated cells displayed an obvious inhibition of microspike formation in a dose‐dependent manner, leading to cell morphology changes such as decreased development of long pseudopodia‐like protrusions; when administered at 20 μmol/L, the microfilament structure was completely destroyed and Golgi organization disruption was also seemingly observed (Figure 7F). We also examined the other main Rho‐GTPase, and only a total of Rac1/2/3 increase accompanied by SKA1 overexpression was observed (Figure 7G).

After cytoskeletal remodelling, cancer cells become more invasive and develop altered affinity to facilitate migration and invasion through basement membrane and ECM, and metastases are triggered subsequently.^25^ Focal adhesions are subcellular structures that mediate the regulatory effects (ie signalling events) of a cell in response to ECM adhesion. Focal adhesion proteins also help hold actin filaments together to form actin stress fibres, which latter are specifically localized to filopodia.^30^, ^31^ We extracted cytoskeletal proteins for immunoblotting experiments, results showed that FAK, Talin and ɑ‐actinin levels increased with SKA1 expression in Capan‐1 cells, FAK, Paxillin and ɑ‐actinin levels decreased with SKA1 knockout in PANC‐1 cells (Figure 7H), preliminarily suggested that SKA1 may be associated with focal adhesions.

Collectively, we drew a simplified schematic diagram illustrating the facilitating effects of SKA1 in actin cytoskeleton organization and related pathways in PDAC cells (Figure 7I).

## 4 DISCUSSION

Early metastasis is quite a challenge to be overcome and is therefore a therapeutic target for preventing the spread of many types of cancer like PDAC.^1^, ^32^

SKA1 was reportedly increased as a candidate oncogene in several common human cancers, contributing to various steps of oncogenesis and poorer prognosis.^14^, ^15^, ^16^, ^17^, ^18^ Not only SKA1 genomic mutations result in chromosome congression failure and subsequent cell changes,^33^ but also SKA1 overexpression itself is primarily responsible for oncogenic function. Besides, SKA1 knock‐down represses proliferation and metastasis in a range of malignancies by regulating the ERK1/2, Akt signalling and cell cycles.^16^, ^17^, ^34^, ^35^ These evidences indicate a multifaceted oncogenic effect driven by SKA1. In this study, we showed that SKA1 was significantly upregulated in PDAC tissues and associated with poor prognosis. Additionally, SKA1 promoted events associated with proliferation and metastatic potential in human PDAC cells in vitro and in vivo. Furthermore, using iTRAQ to screen the differentially expressed proteins affected by SKA1, we demonstrated that SKA1 facilitated Cdc42 activation, thereby prolonging the length of pseudopodia and enhancing cell migration and invasion.

Several compelling lines of evidence indicate that dysregulated cytoskeleton reorganization is a major mechanical drive in EMT and metastasis.^36^, ^37^ Besides, the actin cytoskeleton plays a key role in the entry into mitosis, delaying G2/M phase, leading to death after cell division.^38^ However, due to highly toxic side effects, the therapeutic potential of targeting the actin cytoskeleton remains untapped and incompatible with clinical application.^39^ Increasing evidence indicates that the three cytoskeletal polymers do not exist as separate entities in the cell, but interact to form a complex network and perform various cellular functions. For various cellular processes, cooperation of microtubules with other cytoskeletal elements also contributes to the activation of downstream effector‐related cell behaviours.^40^, ^41^ Here, we demonstrated that SKA1 could regulate actin cytoskeleton remodelling and shared partial co‐localization with F‐actin. Meanwhile, cytochalasin B treatment could abolish the formation of stress fibres and filopodia in PDAC cells, which pattern is similar to that described by other studies.^42^ According to our previous study,^43^ inefficient drug delivery may be an important contributor to chemo‐resistance in pancreatic cancer, we speculate that cytochalasin B may perform enhanced anti‐tumour activity combined with chemotherapy in PDAC. Nevertheless, considering the unacceptable toxicity to normal cells and tissues reported,^39^, ^44^, ^45^ more caution and further investigation are required. Moreover, we demonstrated that Cdc42, which binds to a variety of effector proteins to regulate actin cytoskeleton remodelling and epithelial cell polarization, was strongly involved in SKA1‐induced PDAC progression, and inhibition of Cdc42 may represents a promising strategy for precise cancer therapy.^46^, ^47^, ^48^ In this study, we used ZCL278 as a powerful tool to assess Cdc42 function.^49^, ^50^ We first provided evidence that treatment with ZCL278 of PDAC cells had similar but less powerful effects compared with cytochalasin B, largely by affecting the downstream Arp2/3 complex and N‐WASP, which play critical roles in actin cytoskeleton organization and metastasis in cancer cells.^26^, ^51^, ^52^ Moreover, although we preliminarily confirmed that cell adhesion‐related proteins increased with SKA1 expression, it still remains to be investigated how SKA1 regulates focal adhesion during cell migration in‐depth.

In summary, we comprehensively characterized SKA1, as a oncogene, could constitute a reasonable biomarker and prognostic factor of PDAC. Also, our findings substantiate that SKA1 plays significant roles in facilitating PDAC cells’ proliferation and metastasis by inhibiting G2/M arrest and remodelling actin cytoskeleton via activating Cdc42, which endow the potential of related downstream inhibitors as interference therapeutic targets for PDAC.

## CONFLICT OF INTEREST

The authors declare that they have no competing interests.

## AUTHOR CONTRIBUTION

LFW, TL and QW involved in study design. TL, XL and BX performed the experiments. WW, MPX, YZ and WQ involved in human samples and clinical data collection. JJL, LMW, YTQ and HX involved in data analysis. TL and LFW involved in manuscript preparation. All authors read and approved the final manuscript.

## ETHICAL APPROVAL AND CONSENT TO PARTICIPATE

All PDAC specimens and written informed consents were obtained from Ruijin Hospital, Shanghai Jiao Tong University School of Medicine. The Ethics Committee of Ruijin Hospital approved the study according to the 1975 Declaration of Helsinki. Moreover, all animal experiments were approved by the institutional animal care and use committee of Shanghai Jiaotong University School of Medicine (IACUC approval number: B‐2019‐004).

## Supporting information

Fig S1

Fig S2

Table S1

## Data Availability

All data generated or analysed during this study are included in this published article and its supporting information files.
