# Supplementary material for: SKA1 regulates actin cytoskeleton remodelling via activating Cdc42 and influences the migration of pancreatic ductal adenocarcinoma cells
Source: Cell Prolif. 2020 Mar 30;53(4):e12799. doi: 10.1111/cpr.12799 (PMC7162805; doi:10.1111/cpr.12799)
Supplement: Supplementary file 1 — Fig S1 [file CPR-53-e12799-s001.docx]

**Supporting Information (Figure_S1_SuppInfo)**

**Gene profiling and bioinformatic analyses** **of EMT-related dysregulated mRNAs in pancreatic cancer**

To identify EMT-related dysregulated mRNAs in pancreatic cancer, we firstly downloaded two independent microarray profiling datasets (TGF-β stimulation induced EMT PANC-1 cell line and control group, including GSE23952 and GSE82293), from the GEO database. Furthermore, 114 overlapped genes were consistently dysregulated in both datasets and were used for further GO analysis by DAVID Bioinformatics Resources (https://david.ncifcrf.gov/). Then, TCGA (https://www.cancer.gov/) database were used to confirm their expression significance between cancer and adjacent normal tissues, and higher SKA1 expression significantly correlated with poor survival outcomes in TCGA database (p＜0.01). Finally, SKA1 was selected for further assessment.


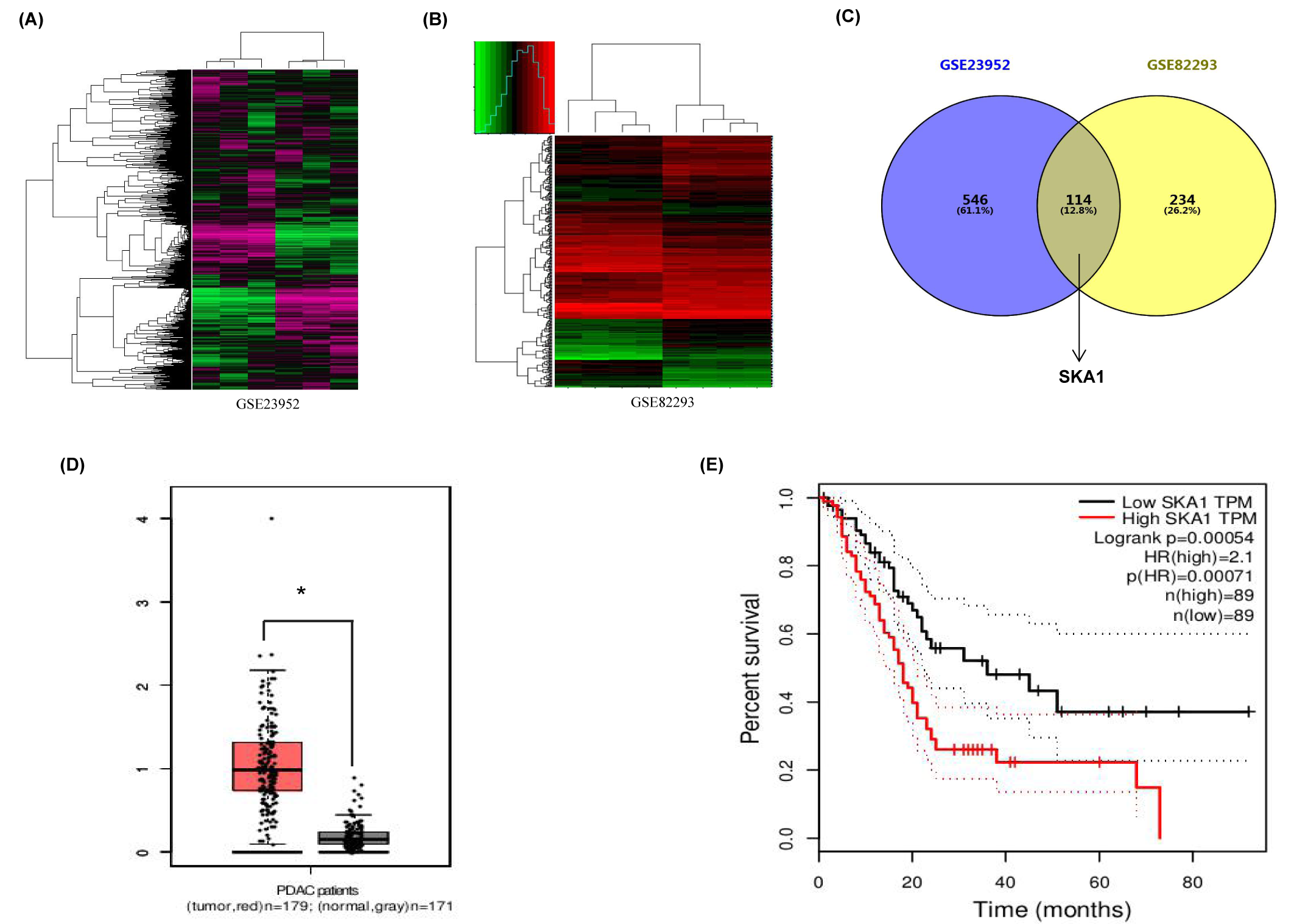


Figure S1 | (A,B) A heatmap was drawn to show the differentially expressed mRNAs in GSE82293 and GSE23952 (TGF-β stimulation induced EMT PANC-1 cell line and control group). Every row represents an individual gene, and each column represents an individual sample. (C) Venn diagram of differentially expressed mRNAs in two datasets. (D) SKA1 mRNA expression was significantly higher in PDAC tumor tissues (n=179) as compared with their adjacent normal tissue (n=171) from TCGA PDAC cancer cohort (p<0.01). (E) Higher SKA1 expression was correlated with poorer survival outcomes in TCGA database (p<0.01).
