# Supplementary material for: SKA1 regulates actin cytoskeleton remodelling via activating Cdc42 and influences the migration of pancreatic ductal adenocarcinoma cells
Source: Cell Prolif. 2020 Mar 30;53(4):e12799. doi: 10.1111/cpr.12799 (PMC7162805; doi:10.1111/cpr.12799)
Supplement: Supplementary file 2 — Fig S2 [file CPR-53-e12799-s002.docx]

**Supporting Information (Figure_S2_SuppInfo)**

**
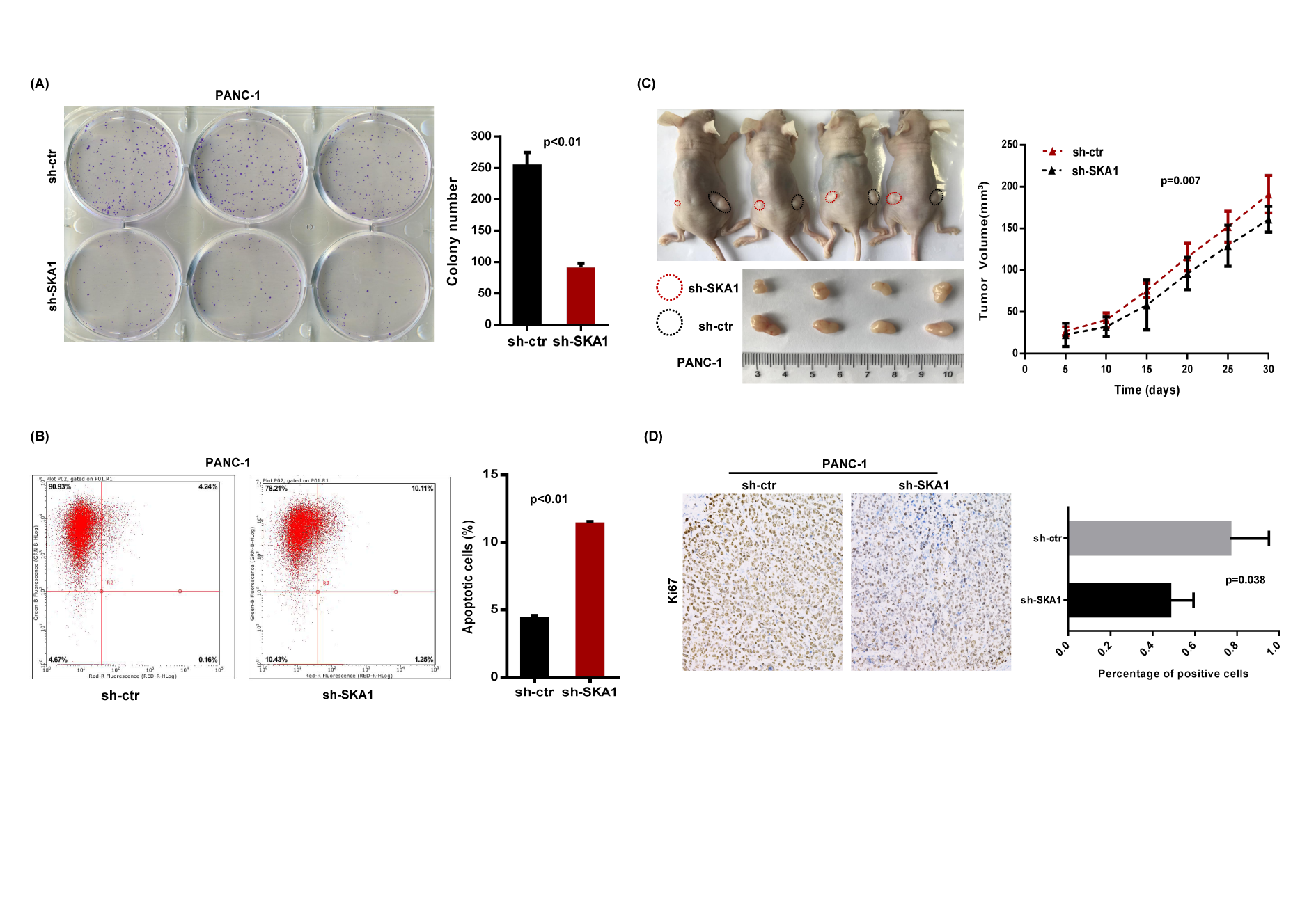
**

**Figure S2 |** Knockdown of SKA1 suppressed PANC-1 cells growth. **(A)** Knockdown of SKA1 suppressed colony formation in PANC-1 cells. **(B)** Knockdown of SKA1 augments apoptosis in PANC-1 cells. **(C)** Knockdown of SKA1 inhibited tumor growth and tumor weight in nude mice (n=4 per group). **(D)** Immunohistochemical analysis of Ki67 in tumor tissues. Percentage of positive Ki67 staining cells was counted.
